# Supplementary material for: Holarctic Species in the Pluteus podospileus Clade: Description of Six New Species and Reassessment of Old Names
Source: J Fungi (Basel). 2023 Aug 31;9(9):898. doi: 10.3390/jof9090898 (PMC10532425; doi:10.3390/jof9090898)
Supplement: Supplementary file 1 [file jof-09-00898-s001.zip › suppl/Supplementary Figure 1 - Bayesian Combined.pdf]

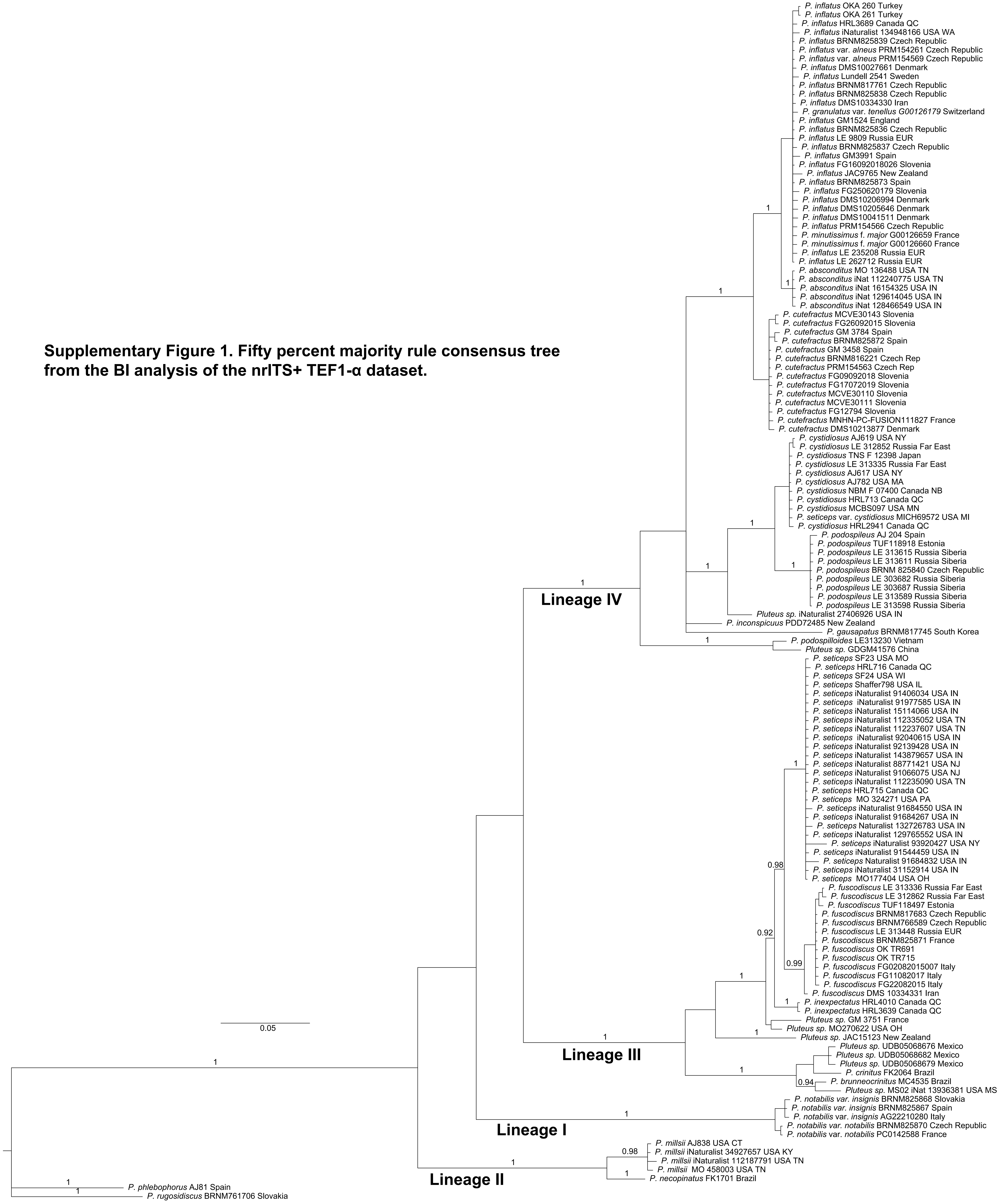

Supplementary Figure 1. Fifty percent majority rule consensus tree from the BI analysis of the nrITS+ TEF1-α dataset.
