# Supplementary material for: Holarctic Species in the Pluteus podospileus Clade: Description of Six New Species and Reassessment of Old Names
Source: J Fungi (Basel). 2023 Aug 31;9(9):898. doi: 10.3390/jof9090898 (PMC10532425; doi:10.3390/jof9090898)

Supplementary Figure 3. Best tree from the ML analysis of the TEF1-α dataset of the /podospileus clade.

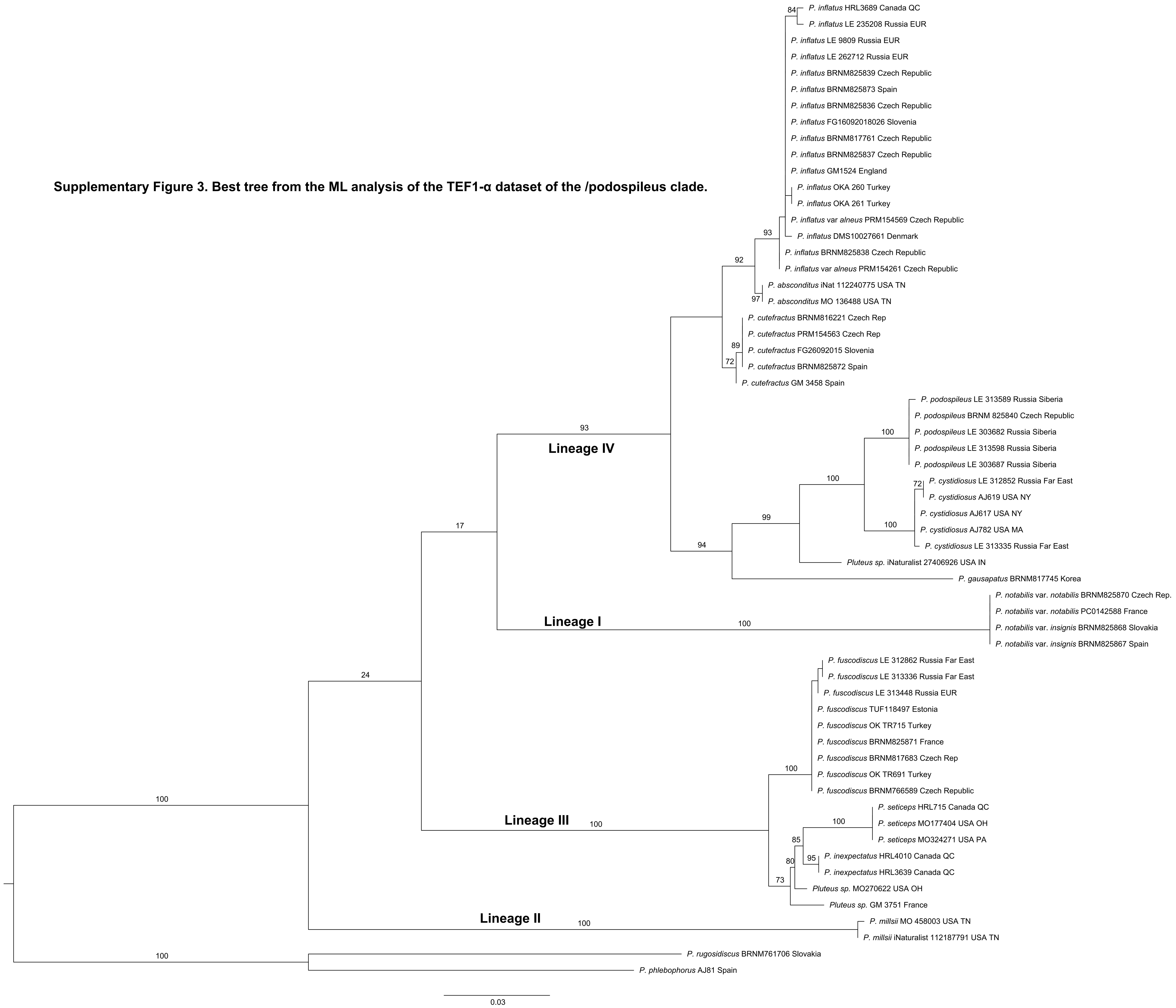

Supplement: Supplementary file 1 [file jof-09-00898-s001.zip › suppl/Supplementary Figure 3 - TEF1 tree.pdf]
